# Low serum cholinesterase predicts complication risk after orthopedic surgery in elderly patients: an observational pilot study

**DOI:** 10.1186/s40981-019-0259-5

**Published:** 2019-06-14

**Authors:** Mitsuhiro Matsuo, Tohru Yamagami

**Affiliations:** 1 Department of Anesthesiology, Itoigawa General Hospital, 457-1 Takegahana, Itoigawa, Niigata 941-8502 Japan; 2 Department of Orthopedic Surgery, Itoigawa General Hospital, Itoigawa, Niigata Japan

**Keywords:** Aged, Frail elderly, Postoperative complications, Preoperative care, Risk assessment

## Abstract

**Background:**

Serum cholinesterase (ChE) in elderly adults is associated with geriatric conditions such as sarcopenia and malnutrition. The aim of this study is to examine the impact of preoperative serum ChE on the development of complications after noncardiac surgery in elderly patients without liver cirrhosis.

**Methods:**

We retrospectively identified all patients aged ≥ 65 years who underwent orthopedic surgery over a 1.5-year period in our hospital. The main outcome was postoperative complications, defined as a deviation from the normal postoperative course within 30 days postoperatively.

**Results:**

A total of 313 patients (median age 79 years) were included. The incidence of all-cause postoperative complications was 15.7% (49/313 patients). Receiver operating characteristic curve analysis showed that serum ChE was a univariable factor that predicted all-cause complications with moderate accuracy (area under the curve = 0.694, 95% confidence interval (CI) 0.604–0.783), with an optimal serum ChE cutoff level of 200 units/L. After multivariate analyses adjusted by baseline characteristics, low serum ChE remained a significant risk factor for postoperative complications (odds ratio = 2.99, 95% CI 1.41–6.33, *P* = 0.004).

**Conclusions:**

Low serum ChE (< 200 unit/L) is a significant risk factor for postoperative complications after orthopedic surgery in patients aged ≥ 65 years.

## Introduction

Pseudocholinesterase is a nonspecific cholinesterase (ChE) that is produced by the liver and distributed to the whole body [1]. Pseudocholinesterase activity is represented by serum ChE in routine diagnostic tests, and serum ChE is a good biomarker for liver cirrhosis [2]. In patients with liver cirrhosis, decreased serum ChE predicts poor survival following cardiac [3, 4] and noncardiac surgery [5, 6]. An increasing number of reports show that serum ChE in elderly adults is associated with geriatric conditions such as sarcopenia [7] and malnutrition [8, 9]. However, little is known about the impact of preoperative serum ChE on the development of postoperative complications in elderly patients without liver disease.

Aging is characterized by a progressive and functional decline of all organ systems, which is associated with an increase in postoperative complications [10]. Thus, advanced age and frailty are independent risk factors for perioperative complications in noncardiac surgery such as orthopedics [10, 11]. Our recent study showed that serum ChE decreases with age in physically independent individuals aged ≥ 65 years [12]. Therefore, we hypothesized that ChE would act as a geriatric marker and predict the development of postoperative complications. To test this hypothesis, we examined the effect of preoperative ChE value on the postoperative complication rate in patients aged ≥ 65 years who underwent orthopedic surgery.

## Methods

### Study design

This retrospective cohort study was approved by the ethics committee of Itoigawa General Hospital (#2018–18, 14 November 2018) and was conducted in accordance with the principles of the Declaration of Helsinki. We included all patients aged ≥ 65 years who underwent orthopedic surgery in our hospital from April 2017 to September 2018. Patients who underwent repeat surgery within 2 months were excluded. ChE activity was determined using p-hydroxybenzoic acid as a substrate with a TBA-c16000 system (Canon Medical System Corp., Otawara, Japan). Serum ChE was routinely evaluated before orthopedic surgery in our hospital. The estimated glomerular filtration rate in milliliters per minute per 1.73 m^2^ was calculated from the creatinine concentration using the Japanese equation [13]. Anemia was defined as hemoglobin of < 10 g/dL. Patients with hypertension, dyslipidemia, and diabetes mellitus requiring medication were included. Patients diagnosed with liver cirrhosis or with a history of hepatocellular carcinoma were defined as having liver failure. End-stage renal disease was defined as an estimated glomerular filtration rate of < 15 mL/min/1.73 m^2^ or the need for dialysis. The main outcome was postoperative complications, defined as a deviation from the normal postoperative course, such as the use of unscheduled antibiotics or a condition requiring specialist investigation within 30 days postoperatively. Major complications were defined as life-threatening diseases.

### Statistical analysis

Data are shown as median [interquartile range]. The optimal cutoff level of ChE was determined by calculating the odds ratio (OR) for every 10 units/L in univariate analysis. Statistical comparisons were performed using the Mann-Whitney test and Fisher’s exact test. A *P* value of < 0.05 was considered statistically significant. Logistic regression analysis was used to adjust all baseline characteristics that yielded *P* values of < 0.05. All statistical analyses were performed using EZR software [14].

## Results

In total, 348 orthopedic surgeries were performed in patients aged ≥ 65 years in our institute over a 1.5-year period. Preoperative serum ChE was not evaluated in 24 patients, and 11 patients were excluded because of reoperation or debridement related to prior surgery. Thus, the remaining 313 patients were included, and the characteristics are summarized in Table 1. All patients were Japanese (median age 79 [72–86] years). No malignant tumor removal was performed during the study period. Although seven patients had liver failure, none had severe cirrhosis classified as Child-Pugh class C. Preoperative ChE in all patients was 267 [221–325] unit/L.

**Table 1:** Characteristics of orthopedic surgery patients in this study

		Postoperative complications		
	Total	Yes	No	*P* value
Number	313	49	264	
Age, years				
65–74 years	95	9	86	
75–89 years	171	26	145	0.011
≥ 90 years	47	14	33	
Gender, female	221	29	192	0.062
BMI, kg/m^2^				
18.5–24.9 kg/m^2^	206	33	173	
< 18.5 kg/m^2^	39	12	27	0.004
> 25.0 kg/m^2^	67	4	63	
Comorbidities				
Anemia	37	10	27	0.054
Hypertension	163	23	140	0.44
Dyslipidemia	88	9	79	0.12
Diabetes mellitus	48	7	41	1
ASA class ≥ 3	104	27	77	< 0.001
Glucocorticoids use	10	2	8	0.66
Organ impairment				
COPD requiring medication	1	0	1	1
History of HF or IHD	25	8	17	0.038
Liver failure	7	1	6	1
ESRD	9	1	8	1
Operative indication				
Upper limb fracture	73	2	71	0.002
Lower limb fracture	137	31	106	
Spinal/pelvic fracture	8	1	7	
Osteoarthritis	28	3	25	
Spinal surgery	28	6	22	
Others	39	6	33	
Surgical procedure				
Operative time ≥ 2 h	58	9	49	1
Bleeding volume ≥ 500 mL	13	3	10	0.44
Emergency	20	3	17	1
Anesthetic procedure				
Spinal	15	3	12	0.71
General	298	46	252	
Postoperative analgesia				
Parenteral analgesics as needed	103	20	83	0.17
Peripheral nerve blocks	151	18	133	
PCA	47	11	36	
PCA combined with nerve blocks	5	0	5	
Epidural analgesia	7	0	7	
Perioperative blood transfusion	68	19	49	0.004

Statistical comparisons were performed using the Fisher’s exact test. *ASA* American Society of Anesthesiologists, *COPD* chronic obstructive pulmonary disease, *DM* diabetes mellitus, *eGFR* estimated glomerular filtration rate, *ESRD* end-stage renal disease, *HF* heart failure, *HTN* hypertension, *IHD* ischemic heart disease, *PCA* patient-controlled analgesia

All-cause complications within 30 days postoperatively developed in 49 of 313 patients (15.7%; Tables 1, 2). The preoperative serum ChE in patients with all-cause complications was significantly lower than that in patients without any complications (222 [178–274] unit/L vs 272 [234–329] unit/L; *P* <  0.001). Two patients (0.6%) died within 30 days postoperatively; they were 80- and 84-year-old women with acute myocardial infarction and preoperative ChE values of 265 and 260 unit/L, respectively. The preoperative serum ChE in patients with major complications was significantly lower than that in patients without major complications (207 [136–260] unit/L vs 267 [223–327] unit/L; *P* = 0.008).

**Table 2:** Complications within 30 days after orthopedic surgery

	*n* = 49
Major complications, *n* = 11	
Heart failure	4
Myocardial infarction	3
Cerebral infarction	2
Pulmonary embolism	2
Minor complications, *n* = 38	
Urinary tract infection	9
Pneumonia	4
Surgical site infection	3
Calcium pyrophosphate deposition disease	3
Deep vein thrombosis in the lower leg	2
Sick sinus syndrome	2
Cellulitis	2
Others (acute kidney injury, bleeding peptic ulcer, cholangitis, etc.)	13

Receiver operating characteristic curve analysis showed that serum ChE predicted all-cause complications as a univariable factor with moderate accuracy (area under the curve = 0.694, 95% confidence interval [CI] 0.604–0.783). The optimal cutoff level of serum ChE was calculated as 200 units/L with a sensitivity of 41.2% and specificity of 86.3%, and the OR was 5.33 (95% CI 2.74–10.4, *P* <  0.001; Table 3). Significant baseline characteristics in Table 1 were used in the logistic regression analysis; age, body mass index, American Society of Anesthesiologists (ASA) class ≥ 3, a history of heart disease, operative indication, and perioperative blood transfusion. After adjustment, low serum ChE remained a significant risk factor for postoperative complications with the OR of 2.99 (95% CI 1.41–6.33, *P* = 0.004; Table 3).

**Table 3:** Impact of low cholinesterase on predicting the development of complications after orthopedic surgery

	Unadjusted		Adjusted	
	OR (95% CI)	*P* value	OR (95% CI)	*P* value
Serum ChE, < 200 unit/L	5.33 (2.74–10.4)	< 0.001	2.99 (1.41–6.33)	0.004

Logistic regression analysis was used to adjust age, body mass index, American Society of Anesthesiologists class ≥ 3, history of heart failure or ischemic heart disease, operative indication, and perioperative blood transfusion. *ChE* cholinesterase, *OR* odds ratio

## Discussion

In the present study, we examined the association between preoperative serum ChE and the development of postoperative complications in orthopedic patients aged ≥ 65 years. After adjustment for possible confounding factors, low serum ChE was a significant risk factor for the occurrence of complications within 30 days postoperatively. These data suggest that consideration of the preoperative serum ChE will enable anesthesiologists and surgeons to improve risk stratification for elderly orthopedic patients.

Serum ChE activity itself is not likely to have a direct effect on the occurrence of postoperative complications. Increasing evidence shows that serum ChE in elderly adult patients is an adverse prognostic factor in conditions other than liver disease, such as bladder cancer [15], coronary artery disease [16, 17], heart failure [18], peripheral artery disease [19], and systemic inflammatory response syndrome [20]. In the present study, multivariate analyses showed that the influence of low serum ChE on postoperative complications did not depend on liver function (Table 3). Serum ChE is associated with frailty conditions, such as sarcopenia [7], malnutrition [8, 9], and body weight loss [21]. The present findings further support the use of ChE as a comprehensive geriatric marker. Additionally, sarcopenia [22] and malnutrition [23, 24] are related to the development of postoperative complications. As low serum ChE is useful as a surrogate marker of geriatric frailty, low serum ChE may be a useful predictor of complications following procedures other than orthopedic surgery in elderly adult patients.

In the present study, the cutoff value of serum ChE was obtained with an optimal OR in univariate analysis. The cutoff level for ChE of 200 units/L was the same as that reported in a previous study that showed that decreased ChE predicts postoperative survival in lung cancer patients with liver cirrhosis [5]. In the present study, the cutoff level for serum ChE of 200 units/L provided a high specificity (86.3%) but low sensitivity (41.2%) in predicting postoperative complications. In fact, the average serum ChE was 204 ± 80 units/L in patients with major complications, and the two dead patients had serum ChE levels of ≥ 200 units/L. Thus, low serum ChE may be a useful marker with which to identify, but not exclude, high-risk patients prior to surgery.

A major limitation of the present study is that no nutritional assessment was performed, although a few patients had severe organ failure that may have caused malnutrition. Moreover, perioperative cognitive function was not assessed [25], as it was difficult to define these diagnoses in this historical medical record review. ChE value is also affected by sarcopenia [7]. However, it was impossible to perform preoperative sarcopenic assessment, such as gait speed and handgrip strength [26], as the reason for surgery was fracture in most patients. In this study, we regarded a deviation from the normal postoperative course as postoperative complications. Although our definition of postoperative complications provided an objective threshold to evaluate in this chart review study, we cannot rule out the possibility that the definition resulted in an overestimation of postoperative complications. Finally, the present study included a small number of patients and was performed in a single institute. The present findings require confirmation in prospective multicenter studies.

## Conclusion

Preoperative low serum ChE predicted the development of complications after orthopedic surgery in elderly adult patients. Further studies are needed to elucidate the precise mechanism by which low serum ChE causes the development of postoperative complications.

## Data Availability

The datasets used and/or analyzed during the current study are available from the corresponding author on reasonable request.

## Abbreviations

ASA: American Society of Anesthesiologists
ChE: cholinesterase
CI: confidence interval
OR: odds ratio
